# Atorvastatin as a Rare Primary Cause of Drug-Induced Angioedema: A Case Report

**DOI:** 10.7759/cureus.28788

**Published:** 2022-09-05

**Authors:** Diana Voloshyna, Saman Al Barznji, Tanveer Ahamad Shaik, Afsar Rizvi, Reya Sachdev, Payal Pritwani, Faraz Saleem, Muhammad Abu Zar Ghaffari

**Affiliations:** 1 College of Medicine, University of Michigan, Ann Arbor, USA; 2 Internal Medicine, University of Sulaymaniyah, Sulaymaniyah, IRQ; 3 Cardiovascular Medicine, University of Louisville School of Medicine, Louisville, USA; 4 General Practice, Swan Practice, Manchester, GBR; 5 Medicine, Ghulam Muhammad Mahar Medical College, Sukkur, PAK; 6 Internal Medicine, Ghulam Muhammad Mahar Medical College, Sukkur, PAK; 7 Internal Medicine, Akhtar Saeed Medical and Dental College, Lahore, PAK

**Keywords:** angioedema, naranjo scale, cardiovascular prevention, cardiovascular, atorvastatin induced angioedema, rare adverse effect, drug-induced angioedema, statin

## Abstract

In patients with hyperlipidemia and cardiovascular disease, statin remains the primary medication for risk reduction. Statins are primarily associated with adverse outcomes like myoglobinuria and deranged liver function tests (LFTs). Angioedema is a life-threatening reaction characterized by mucosal and submucosal swelling. It is rarely known for its association with statins. However, we present a rare case of a 59-year-old man presenting with recurrent angioedema of the face and tongue after starting on 40mg of atorvastatin, within one week of the treatment. He had no previous history of hypersensitivity and rash. He denied any food or medication allergy in the past. The Naranjo scale probability and the abrupt nature of these episodes upon starting statin and completely resolving after discontinuing the drug made statin-induced angioedema the primary diagnosis in this case.

## Introduction

Angioedema involves the swelling of mucosa and submucosal tissue. It generally manifests as the edema of the face, lips, and tongue. It may be severe and fatal as it may progress to involve the respiratory tract [1]. Hydroxymethyl glutaryl coenzyme A (HMG Co-A) reductase inhibitors are one of the first-line drugs for lowering the serum levels of cholesterol [2]. Although these drugs have a good safety profile, severe adverse effects can occur, including backache, gastrointestinal upset, myositis, and elevated liver enzymes [2,3]. After food, medicine is considered the most prevalent cause of angioedema presenting to the emergency department [1]. It is estimated that around 32% of occurrences of angioedema are caused by medications [3]. No such cases have ever been reported for atorvastatin. We hereby discuss the case of a 59-year-old male with occurrences of self-resolving edema of the face, lips, and tongue while taking atorvastatin. 

## Case presentation

A 59-year-old Asian male patient with a family history of coronary heart disease and high cholesterol presented to the primary care physician to get his recently diagnosed hyperlipidemia evaluated. The patient was healthy, non-hypertensive, and non-diabetic and was not taking any medications. The patient had no history of allergies to drugs or food substances and no adverse hypersensitivity reactions in the past. After a detailed examination and history, the patient was started on atorvastatin 40mg for his hyperlipidemia and was advised to begin a heart-healthy diet and regular exercise program. 

**Figure 1:**
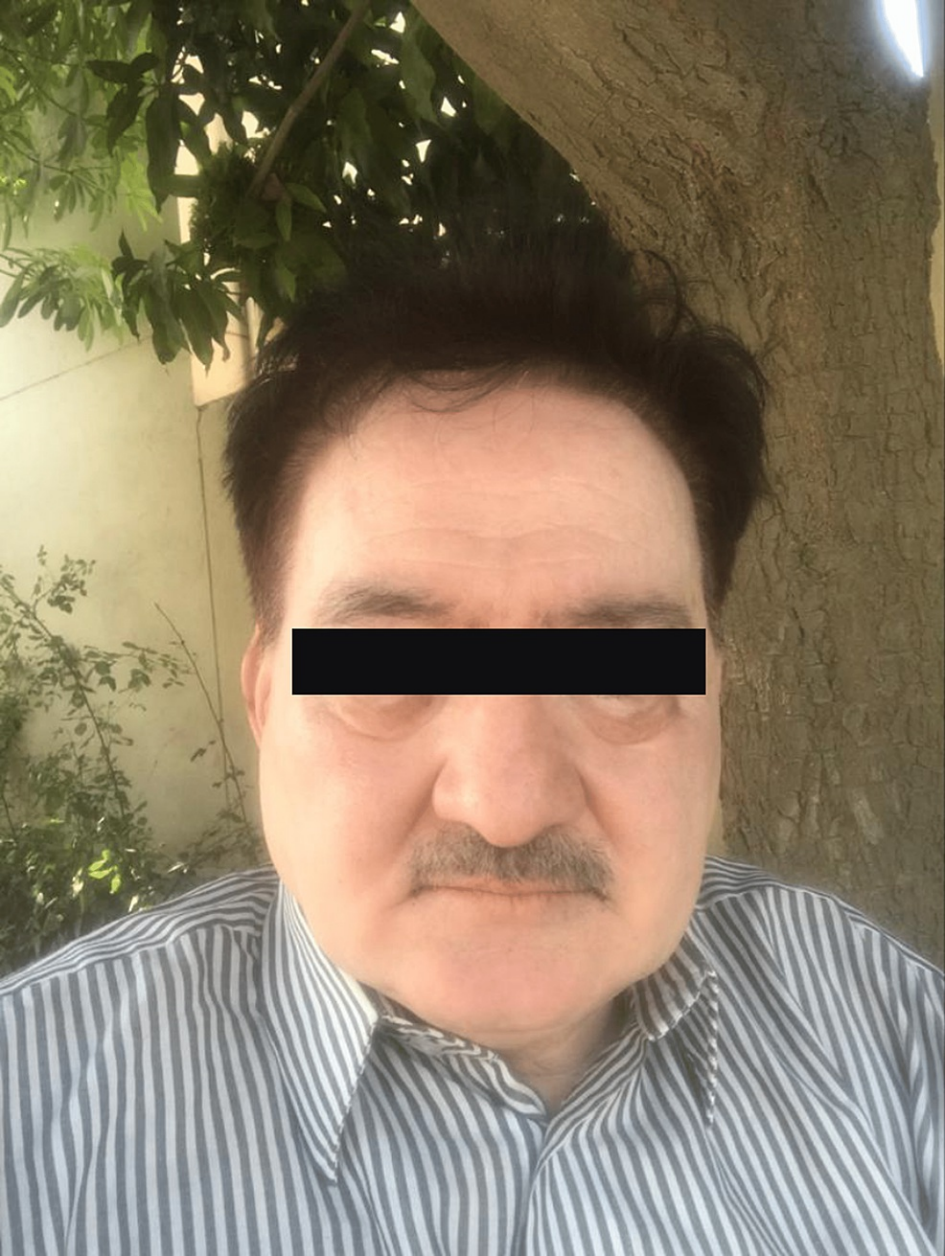
Atorvastatin-induced angioedema

Shortly after starting the treatment, the patient noticed numbness and swelling of the tongue and face that occurred within a few hours of statin intake; despite this, he continued taking the statin for a few days, and these episodes kept on recurring with increased severity (Figure 1).

**Figure 2:**
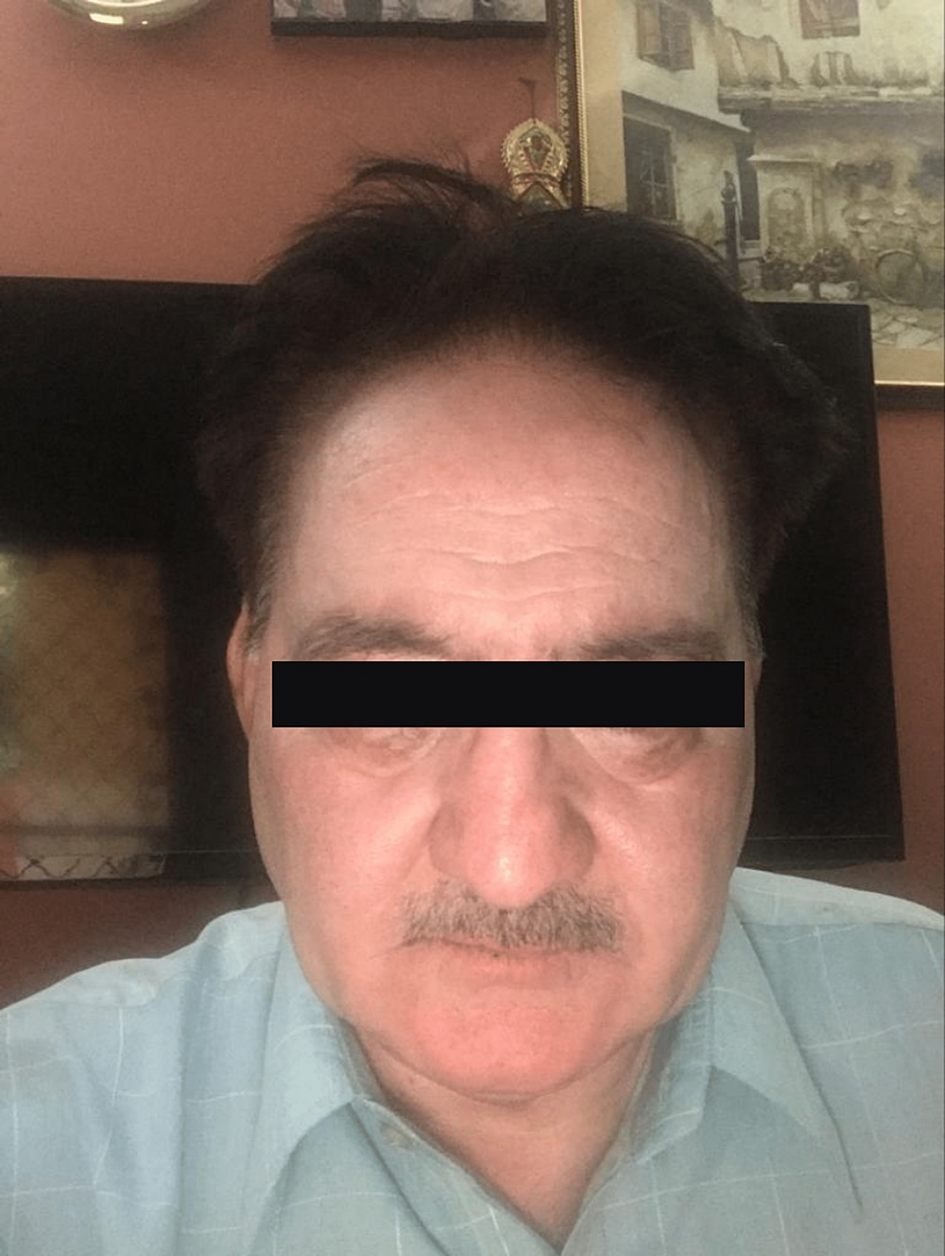
Patient twenty-four hours after discontinuing atorvastatin

Consequently, the patient was rushed to our clinic for further evaluation. On examination, his face was swollen and numb, and he had no rash or itchiness; his tongue was swollen mildly, but he had no problem breathing and swallowing. The patient was hemodynamically stable. A provisional diagnosis of angioedema was made, and IV prednisolone was given. The swelling subsided over the next 24 hours without the use of any additional corticosteroid therapy (Figure 2).

The patient had no such history of swelling and adverse hypersensitivity reactions in the past.

Moreover, the patient had not eaten anything unusual and had no recent travel history or food allergy. Laboratory findings were unremarkable, and the eosinophilic count was normal. The patient's drug history was reviewed, and statin was found to be the primary cause for such recurrent angioedema, as these episodes occurred only after oral statin intake. Based on the Naranjo Adverse Drug Reaction Probability Scale, a score of 6 was observed, showing probable cause (Table 1).

**Table 1:** Naranjo Adverse Drug Reaction Probability Scale showing a score of 6 (probable cause) [4]

Questions	Yes	No	Do not know	Score
1. Are there previous conclusive reports on this reaction?	1	0	0	1
2. Did the adverse event appear after the suspected drug was administered?	2	-1	0	2
3. Did the adverse reaction improve when the drug was discontinued or a specific antagonist was administered?	1	0	0	1
4. Did the adverse event reappear when the drug was re‐administered?	2	-1	0	2
5. Are there alternative causes (other than the drug) that could on their own have caused the reaction?	-1	2	0	-1
6. Did the reaction reappear when a placebo was given?	-1	1	0	1
7. Was the drug detected in blood (or other fluids) in concentrations known to be toxic?	1	0	0	0
8. Was the reaction more severe when the dose was increased or less severe when the dose was decreased?	1	0	0	0
9. Did the patient have a similar reaction to the same or similar drugs in any previous exposure?	1	0	0	0
10. Was the adverse event confirmed by any objective evidence?	1	0	0	1
Total score :				6

As a result, a diagnosis of statin-induced angioedema was made. The patient was started on a different class of drug (ezetimibe) for his hyperlipidemia. On his one-week follow-up, he reported no such episodes.

## Discussion

Angioedema can be caused by the release of histamine following mast cell degranulation or by an increase in bradykinin accumulation due to either an increase in production or a reduction in inactivation [3]. As more medicines linked to angioedema have been found, other ideas about how drugs might cause angioedema have come to light. Numerous processes enhance vascular permeability, resulting in subcutaneous fluid buildup. There is currently insufficient evidence to determine whether statins cause angioedema [5].

Drug-induced angioedema is most commonly linked to angiotensin-converting enzyme (ACE) inhibitors [2]. Several additional drugs, including calcium channel antagonists, thienopyridines, and hydrochlorothiazide, have been linked to drug-induced angioedema [5]. Statin-induced angioedema may be mediated by bradykinin, which causes vasodilation and potentially angioedema [3].

Angioedema caused by drugs can be either allergic or non-allergic. Drug-induced angioedema is a type I hypersensitivity reaction, with histamine playing a key role in the reaction. It presents itself with a rapid onset of urticarial rash and edema of the mucosal and submucosal tissues. Treatment with antihistamines, epinephrine, and corticosteroids alleviates symptoms rapidly [5].

Non-allergic angioedema triggered by drugs is mostly mediated by bradykinin. Unlike histamine-mediated angioedema, this kind of angioedema develops gradually over time. When treated with antihistamines and corticosteroids, symptoms may improve within two to five days. The resolution of medication-induced non-allergic angioedema occurs when the drug is discontinued [1]. It is not known if angioedema is dosage dependent or related to medication concentrations in the serum [5].

At least two pathways have been found in non-allergic angioedema produced by statins. Initially, statins increase the number of bradykinin type 2 receptors that are present on endothelial cells. In an experiment involving the growth of human coronary endothelial cells, lovastatin increased bradykinin type two receptors. The effect of bradykinin on its receptors may potentially be amplified by statins. By increasing the release of nitric oxide and prostacyclin, these mechanisms might enhance a patient's susceptibility to developing angioedema in the presence of circulating bradykinin [6,7]. Rosuvastatin and angioedema have been shown to have a definitive connection, according to the Naranjo likelihood scale [4]. 

Pre-existing postmarketing reports, incidence following rosuvastatin injection, and improvement following statin discontinuation were all positively associated [2]. The combination of an increase in bradykinin type 2 receptors caused by statin usage and activation of the kinin system caused by amlodipine may hasten the onset of angioedema. Based on this suggested mechanism, it is likely that statin usage may predispose individuals to angioedema in the presence of another triggering condition that raises circulation bradykinin [5]. 

The onset of these responses might vary between two days and nine months. There are no laboratory-based diagnostics that give quick therapy suggestions. C4 levels are consistently diminished in hereditary angioedema. Tryptase levels are higher in patients with allergic edema. If there is no tongue or laryngeal involvement or any evidence of airway compromise, the patient can be discharged after 12 to 24 hours of surveillance [2]. In severe cases of angioedema caused by histamine, the standard treatment of corticosteroids and antihistamines may be combined with epinephrine. Fresh frozen plasma is currently the only effective acute therapy for bradykinin-mediated angioedema [8].

The Food and Drug Administration (FDA) has approved specific medications such as icatibant, ecallantide, and C1-inhibitor concentrate for the treatment of acute histamine-mediated angioedema attacks. There is some evidence that they help in drug-induced angioedema, but not enough to recommend them for patients without hereditary angioedema [8]. Negative reactions to medications have far-reaching consequences for patient care since they eliminate not only the offending drug but all others in the same pharmacological class. Adverse medication reactions are rarely specific; diagnostic testing is frequently unavailable; drug reintroduction is not morally justifiable [9].

## Conclusions

Drug-induced side effects may indicate the need for a comprehensive evaluation of all the patients' medications. Statins cannot be disregarded as the primary or contributing cause of angioedema. The immediate withdrawal of the suspected drug(s) and treatment with antihistamines, corticosteroids, and epinephrine should be the primary goal to prevent the severity of angioedema.
